# Polymorphisms in folate-metabolizing genes, chromosome damage, and risk of Down syndrome in Italian women: identification of key factors using artificial neural networks

**DOI:** 10.1186/1755-8794-3-42

**Published:** 2010-09-24

**Authors:** Fabio Coppedè, Enzo Grossi, Francesca Migheli, Lucia Migliore

**Affiliations:** 1 Department of Human and Environmental Sciences, Section of Medical Genetics, University of Pisa, Italy; 2 Bracco Medical Department, San Donato Milanese, Italy; 3 Semeion Research Centre, Rome, Italy

## Abstract

**Background:**

Studies in mothers of Down syndrome individuals (MDS) point to a role for polymorphisms in folate metabolic genes in increasing chromosome damage and maternal risk for a Down syndrome (DS) pregnancy, suggesting complex gene-gene interactions. This study aimed to analyze a dataset of genetic and cytogenetic data in an Italian group of MDS and mothers of healthy children (control mothers) to assess the predictive capacity of artificial neural networks assembled in TWIST system in distinguish consistently these two different conditions and to identify the variables expressing the maximal amount of relevant information to the condition of being mother of a DS child.

The dataset consisted of the following variables: the frequency of chromosome damage in peripheral lymphocytes (BNMN frequency) and the genotype for 7 common polymorphisms in folate metabolic genes (*MTHFR *677C>T and 1298A>C, *MTRR *66A>G, *MTR *2756A>G, *RFC1 *80G>A and *TYMS *28bp repeats and 1494 6bp deletion). Data were analysed using TWIST system in combination with supervised artificial neural networks, and a semantic connectivity map.

**Results:**

TWIST system selected 6 variables (BNMN frequency, *MTHFR *677TT, *RFC1 *80AA, *TYMS *1494 6bp +/+, *TYMS *28bp 3R/3R and *MTR *2756AA genotypes) that were subsequently used to discriminate between MDS and control mothers with 90% accuracy. The semantic connectivity map provided important information on the complex biological connections between the studied variables and the two conditions (being MDS or control mother).

**Conclusions:**

Overall, the study suggests a link between polymorphisms in folate metabolic genes and DS risk in Italian women.

## Background

**Figure 1:**
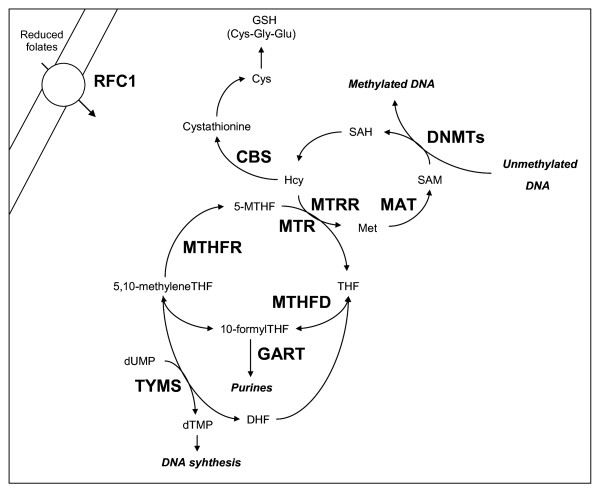
**Overview of the folate metabolic pathway**. Folates require several transport systems to enter the cells, the best characterized being the reduced folate carrier (RFC1). Methylenetetrahydrofolate reductase (MTHFR) reduces 5,10-methylenetetrahydrofolate (5,10-MTHF) to 5-methyltetrahydrofolate (5-MTHF). Subsequently, methionine synthase (MTR) transfers a methyl group from 5-MTHF to homocysteine (Hcy) forming methionine (Met) and tetrahydrofolate (THF). Methionine is then converted to S-adenosylmethionine (SAM) in a reaction catalyzed by methionine adenosyltransferase (MAT). Most of the SAM generated is used in transmethylation reactions, whereby SAM is converted to S-adenosylhomocysteine (SAH) by DNA methyltransferases (DNMTs) that transfer the methyl group to the DNA. Vitamin B12 (or cobalamin) is a cofactor of MTR, and methionine synthase reductase (MTRR) is required for the maintenance of MTR in its active state. If not converted into methionine, Hcy can be condensed with serine to form cystathionine in a reaction catalyzed by cystathionine β-synthase (CBS), which requires vitamin B6 as a cofactor. Cystathionine can be then utilized to form the antioxidant compound glutathione (GSH). Another important function of tetrahydrofolate derivatives is in the de novo synthesis of DNA and RNA precursors, where they are used by thymidylate synthase (TYMS) and methylenetetrahydrofolate dehydrogenase (MTHFD) for the synthesis of nucleic acid precursors. MTHFD is a trifunctional enzyme that interconverts tetrahydrofolate derivatives for purine, methionine and thymidylate synthesis. TYMS requires 5,10-MTHF and deoxyuridine monophosphate (dUMP) for the production of to deoxythymine monophospate (dTMP) and dihydrofolate (DHF) in the de novo synthesis of pyrimidines. Other enzymes participate in folate metabolism, among them phosphoribosylglycinamide transformylase (GART) which is a protein required for purine synthesis.

Folates are essential nutrients required for one-carbon biosynthetic and epigenetic processes. After intestinal absorption, folate metabolism requires reduction and methylation into the liver to form 5-methyltetrahydrofolate (5-methylTHF), release into the blood and cellular uptake; then it can be used for the synthesis of DNA and RNA precursors or for the conversion of homocysteine (Hcy) to methionine, which is then used to form the main DNA methylating agent S-adenosylmethionine (SAM) [1,2]. A diagram illustrating folate metabolism is shown in figure 1. Deficiencies in cellular folates result in aberrant DNA methylation, point mutations, chromosome breakage, defective chromosome recombination and aneuploidy [3]. Therefore, in 1999 James and coworkers suggested that impairments in folate metabolism due to genetic polymorphisms of metabolic enzymes could increase the risk for having an infant with Down syndrome (DS) [4]. That paper stimulated considerable investigation into the possible role of folate metabolism in the risk of having a DS child and we recently reviewed all the genetic association studies performed from 1999 to 2009 [2]. Few additional papers have been produced after the publication of that review [5-8] for a total of almost 30 research articles available in Pubmed and aimed at addressing this issue. However, despite considerable researches in the field, results are often conflicting or inconclusive and the question is still unsolved [2,9,10]. The current opinion is that the combined presence of several polymorphisms in the folate metabolic pathway in interaction with environmental factors might increase the risk for a DS pregnancy, rather than the presence of single polymorphic variants alone. However, the major limit of all the studies performed so far is the small sample size of the case-control groups which largely reduces the statistical power to test for genetic associations and for gene-gene and gene-environment interactions by means of traditional statistical approaches, such as logistic regression analyses [2]. Given the complexity of the folate metabolic pathway and the number of genes and environmental factors involved, we have estimated that the design of a case-control study able to test the contribution of each of these factors to DS risk with adequate power would require several thousands individuals [2]. Unfortunately, all the genetic association studies so far have been performed in groups of 100-200 mothers of DS individuals (MDS) or less, making it impossible to come to a definitive conclusion [2].

We observed that MDS who had a DS child in young age (35 years or before) have an increased frequency of chromosome damage and malsegregation events in peripheral blood lymphocytes, detectable by means of the micronucleus assay coupled by fluorescence in situ hybridization technique compared to women who gave birth to healthy children [11]. Others have recently suggested that women who have a DS child in young age might have an increased tendency to chromosome malsegregation events beginning from the first embryonic mitotic divisions of their body [12]. Subsequent studies by us revealed association between folate gene polymorphisms and the frequency of binucleated micronucleated lymphocytes (BNMN‰) in blood from MDS and control mothers, providing an easily accessible biomarker (BNMN frequency) that can be measured to link impaired folate metabolism to chromosome malsegregation [13,14].

Overall, genetic association studies as well as cytogenetic studies performed in MDS point to a possible role of folate gene polymorphisms in affecting chromosome malsegregation events, thus increasing the risk for a DS pregnancy. Moreover, they all suggest complex interactions between several factors within the folate metabolic pathway [2].

We performed the present study using Artificial Neural Networks (ANNs) to identify key factors linking folate metabolism to chromosome malsegregation and the risk of having a DS child. The method used by ANNs aims to understand natural processes and recreate those processes using automated models. These networks allow a method of forecasting with understanding of the relationship between variables, in particular non-linear relationships [15-17]. ANNs function by initially learning a known set of data from a given problem with a known solution (training) and then the networks, inspired by the analytical processes of the human brain, are able to reconstruct the imprecise rules which may be underlying a complex set of data (testing). In recent years ANNs have been used successfully in medicine, for example they have been used to investigate the predictive values of risk factors on the conversion of amnestic mild cognitive impairment to Alzheimer's disease [18], to identify placental determinants of fetal growth [19], to identify genetic variants essential to differentiate sporadic amyotrophic lateral sclerosis cases from controls [20], and to distinguish between Alzheimer's disease patients and controls [21], among others.

This study aimed to analyze a dataset of genetic and cytogenetic data obtained from MDS and mothers of healthy children (control mothers) [13,14] to assess the predictive capacity of artificial neural networks assembled in TWIST system [22] in distinguish consistently these two different conditions and to identify the variables expressing the maximal amount of relevant information to the condition of being mother of a DS child. A series of supervised multilayer perceptrons with four hidden units were then employed to validate the choice of variables made by TWIST system. Moreover, we constructed a semantic connectivity map to offer some insight regarding the complex biological connections between the studied variables and the two conditions (being MDS or control mother).

## Methods

### Database

We aimed to re-analyze from a completely new perspective most of the data obtained from our previous studies [13,14]. From a previously described database [13,14] containing data from MDS (35 years or less; range 19-35) that gave birth to a DS child and data from control mothers (35 years or less; range 20-35) that gave birth to healthy children and had no miscarriages or complications during pregnancies, we have selected 29 MDS and 32 control mothers for whom all the following information was available: 1) BNMN frequency, 2) genotype for the *MTHFR *677C>T polymorphism (CC, CT or TT), 3) genotype for the *MTHFR *1298A>C polymorphism (AA, AC or CC), 4) genotype for the *MTRR *66A>G polymorphism (AA, AG or GG), 5) genotype for the *MTR *2756A>G polymorphism (AA, AG or GG), 6) genotype for the *RFC1 *80G>A polymorphism (GG, AG, AA), 7) genotype for *TYMS *28bp repeats (2R2R, 2R3R,3R3R) and 1494 6bp deletion (+/+, +/-, -/-) polymorphisms. Table 1 shows the distribution of these variables among MDS and control mothers. As detailed elsewhere [13,14] all MDS and control mothers included in the database were Caucasians of Italian origin (Tuscany and neighbouring areas). Concerning MDS, full trisomy 21 of the children (primary trisomy) was confirmed by cytogenetic analysis. Control mothers had no miscarriages or children affected by genetic disorders in their life, and at least one healthy child before age 35 years; they were recruited by us among women employed in the University Hospital of Pisa. All the subjects included in the study were matched for age either at time of delivery and at sampling (Table 2). The individuals included in the database have been selected after the administration of a detailed questionnaire, designed to document their previous conditions, dietary habits, smoking habits, working environment and pharmacological treatments in order to exclude those subjects exposed to environmental factors known to interfere with the BNMN frequency. Additional information can be found in our previous publications [13,14].

**Table 1:** Distribution of studied variables among MDS and control mothers

Variable	MDS (n = 29)	Controls (n = 32)
*MTHFR *677C>T	CC: 5	CC: 11
	CT: 19	CT: 17
	TT: 5	TT: 4
		
*MTHFR *1298 A>C	AA: 14	AA: 13
	AC: 15	AC: 19
	CC: 0	CC: 0
		
*MTRR *66 A>G	AA: 9	AA: 12
	AG: 14	AG: 14
	GG: 6	GG: 6
		
*MTR *2756 A>G	AA: 20	AA: 24
	AG: 9	AG: 6
	GG: 0	GG: 2
		
*RFC1 *80 G>A	GG: 12	GG: 10
	GA: 16	GA: 12
	AA: 1	AA: 10
		
*TYMS *28bp repeat	2R/2R: 5	2R/2R: 6
	2R/3R: 19	2R/3R: 14
	3R/3R: 5	3R/3R: 12
		
*TYMS *1494 6bp deletion	6bp +/+: 5	6pb +/+: 8
	6bp +/-: 21	6bp +/-: 19
	6bp -/-: 3	6bp -/-: 5
		
BNMN‰ (mean ± SD)	16.5 ± 7.6	9.3 ± 3.1

**Table 2:** Demographic characteristics of the study population

Study group	Number of subjects	Age at delivery **mean ± S.D**.	Age at sampling **mean ± S.D**.
MDS	29	28.2 ± 4.7	49.6 ± 11.2

Control mothers	32	28.5 ± 5.2	47.3 ± 6.9

**Figure 2:**
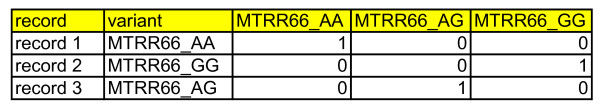
**Method of coding the polymorphisms in the database**. The code assigned to the polymorphisms transformed each polymorphism in three genotype classes: major homozygous, heterozygous and minor homozygous. For each class a binary coding was applied: 0 if variable absent; 1 if variable present. So for example considering the polymorphism *MTRR *66A>G which can exist in three variants: AA (major homozygous), AG (heterozygous) and GG (minor homozygous). Supposing that three records are AA, GG and AG, the coding has been applied as shown in the figure.

The database analyzed in the present study is provided as additional material (see additional file 1: database, .csv format). Figure 2 explains how genotypes were coded in the database.

### Genotyping and BNMN frequency

The database data concerning the BNMN frequency and the genotype for all the 7 studied polymorphisms had been previously obtained by means of the cytokinesis-block micronucleus assay (BNMN frequency) and validated PCR/RFLP techniques as described elsewhere [13,14]. All the samples were coded and data were processed by blinded operators. All individuals gave informed consent for inclusion in the database, whose creation was performed in accordance with the Helsinki Declaration and approved by the "Stella Maris" I.R.C.C.S. Ethics Committee as described elsewhere [13,14].

### Artificial neural networks analysis

Advanced intelligent systems based on novel coupling of artificial neural networks and evolutionary algorithms have been applied. In this study we applied TWIST system [22] and supervised ANNs in order to develop a model able to predict with high degree of accuracy the diagnostic class starting from genotype data alone. Supervised ANNs are networks which learn by examples, calculating an error function during the training phase and adjusting the connection strengths in order to minimize the error function [23]. The learning constraint of the supervised ANNs makes their own output coincide with the predefined target. The general form of these ANNs is: y = f(x, w*), where w* constitutes the set of parameters which best approximate the function.

### TWIST system

Data analysis was performed using a re-sampling system named TWIST developed by Semeion Research Centre. The TWIST system consists in an ensemble of two previously described systems: T&T and IS [22]. The T&T system is a robust data re-sampling technique that is able to arrange the source sample into sub-samples that all possess a similar probability density function. In this way, the data is split into two or more sub-samples in order to train, test and validate the ANN models more effectively. The IS system is an evolutionary wrapper system able to reduce the amount of data while conserving the largest amount of information available in the dataset. The combined action of these two systems allow us to solve two frequent problems in managing Artificial Neural Networks, i.e. the optimal splitting of the data set in training and testing subsets containing a balanced distribution of outliers and the optimal selection of variables with maximal amount of information relevant to the problem under investigation. Both systems are based on a Genetic Algorithm, the Genetic Doping Algorithm (GenD) developed at Semeion Research Centre [24]. The TWIST system has been previously applied in different medical contexts [25], additional data are given (see additional file 2: Twist System, pdf file).

After this processing, the features that were most significant for the classification were selected and at the same time the training set and the testing set were created with a function of probability distribution similar to the one that provided the best results in the classification. A series of supervised Multi Layer Perceptrons, with four hidden units, were then used for the classification task. The final ANNs which were trained and tested on the new data set generated by TWIST system are "virgin" and operate independently and blindly from each other and from TWIST system.

### Semantic connectivity map

An existing mapping method [26,27] was used to highlight through a graph the most important links among variables, using a mathematical approach based on an artificial adaptive system called Auto Contractive Map-Auto-CM algorithm. The Auto Contractive Map (Auto-CM) is a special kind of Artificial Neural Network able to find, by a specific data mining learning algorithm, the consistent patterns and/or systematic relationships and hidden trends and associations among variables. After the training phase the weights developed by Auto-CM are proportional to the strength of associations of all variables each-other. The weights are then transformed in physical distances. Variables couples whose connection weights are higher become nearer and vice versa. A simple mathematical filter represented by minimum spanning tree is applied to the distances matrix and a graph is generated. This allows seeing connection schemes among variables and detecting variables acting ad "hubs", being highly connected. This matrix of connections preserves non linear associations among variables and captures connection schemes among clusters.

After the training phase, the weights matrix of the Auto-CM represents the warped landscape of the dataset. Subsequently, a simple filter to the weights matrix of the Auto-CM system was applied to obtain a map of the main connections between the variables of the dataset and the basic semantic of their similarities, defined connectivity map as detailed by Buscema and Grossi [26] and Buscema et al. [27]. The theory behind Auto-CM system is provided as additional material (see additional file 3: Auto-CM System, pdf file).

## Results

### Classification performances with ANNs

The linear correlation index between the input variables and the target variable was generally very low, with exception of BNMN‰ (r = 0.54). This gave the rationale to employ artificial neural networks.

**Figure 3:**
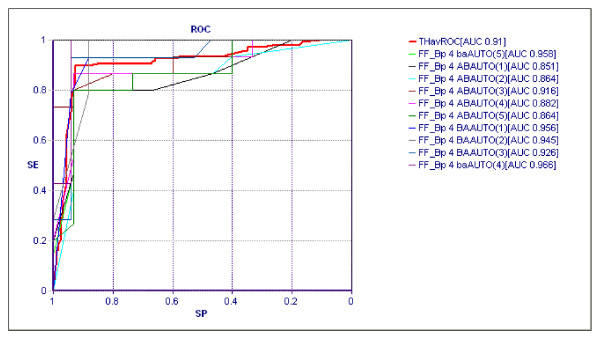
**Area Under the Curve (AUC) of Receiver Operator Characteristic (ROC) relative to ten classification tasks performed with Back Propagation ANN models**. The ROC represents therelationship between sensitivity and specificity for the prediction of each of the considered outcomes. The average ROC is depicted in red

The application of TWIST system allowed the selection of a subgroup of 6 variables described in table 3. This new data set has been analyzed with Back propagation ANNs employing a rigorous validation protocol. The validation protocol is a procedure to verify the models' ability to generalize the results reached in the testing phase. Among the different protocols reported in literature, the selected model is the protocol with the greatest generalization ability on data unknown to the model itself. The procedural steps in developing the validation protocol are: 1) subdividing the dataset randomly into two sub-samples: the first called Training Set, and the second, called Testing Set; 2) choosing a fixed ANN (and/or Organism) which is trained on the Training Set. In this phase, the ANNs learns to associate the input variables with those that are indicated as targets; 3) saving the weight matrix produced by the ANNs at the end of the training phase, and freezing it with all of the parameters used for the training; 4) showing the Testing Set to the ANNs, so that in each case, the ANNs can express an evaluation based on the training just performed. This procedure takes place for each input vector but every result (output vector) is not communicated to the ANNs; in this way, the ANNs are evaluated only in reference to the generalization ability that it has acquired during the Training phase; 5) constructing a new ANN with identical architecture to the previous one and repeating the procedure from point 1. This general training plan has been employed five times with obtaining 10 independent classification experiments. We have employed the so called 5x2 cross-validation protocol [28] which produces 2 elaborations for every sample, the first with training on subset a and testing on subset b and the second with training on subset b and testing on subset a. Table 4 summarizes the results obtained with back propagation ANN applied ten times on the final data set. Figure 3 shows the Area Under the Curve (AUC) of Receiver-Operating Characteristic (ROC) AUC of the ten classifications and the average ROC AUC in red.

**Table 3:** Variables selected by TWIST system

	Selected Variables
1	*MTR *2756 AA
2	*MTHFR *677TT
3	*RFC1 *80 AA
4	*TYMS *1494 6bp +/+
5	*TYMS *28bp 3R/3R
6	BNMN ‰

**Table 4:** Classification performances of back propagation neural networks on final data set

ANN	Sensitivity	Specificity	Global accuracy	ROC AUC
Back propagation 1(a-b)	80	93,33	86,67	0,864
Back propagation 2(a-b)	80	93,33	86,67	0,851
Back propagation 3(a-b)	80	93,33	86,67	0,864
Back propagation 4(a-b)	80	93,33	86,67	0,916
Back propagation 5(a-b)	86,67	93,33	90	0,882
Back propagation 1(b-a)	100	94,12	97,06	0,958
Back propagation 2(b-a)	100	88,24	94,12	0,956
Back propagation 3(b-a)	100	88,24	94,12	0,945
Back propagation 4(b-a)	92,86	94,12	93,49	0,926
Back propagation 5(b-a)	100	94,12	97,06	0,966
**Mean**	**89,95**	**92,55**	**91,25**	**0,91**

### Semantic connectivity map

**Figure 4:**
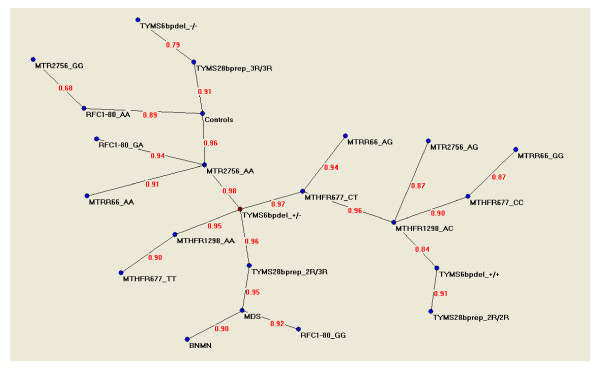
**Semantic connectivity map obtained with Auto-Cm System**. The figures on the arches of the graph refer to the strength of the association Between two adjacent nodes. The range of this value is from 0 to 1.

Figure 4 shows the semantic connectivity map obtained with the application of Auto-CM system. Variables which have the maximal amount of connections with other variables are called "hubs" of the system. In order to better understand the meaning of the connections a numerical value is applied to each edge of the graph. This value, deriving from the original weight developed by Auto-CM during the training phase scaled from 0 to 1, is proportional to the strength of the connections among two variables.

The *TYMS *1494 6bp +/- genotype resulted to be the principal hub of the system, i.e. the dominant variable. This variable was connected with *TYMS *28bp 2R/3R, with *MTR *2756AA, and with both *MTHFR *677CT and *MTHFR *1298AA variables. Connections were detected among *MTHFR *677 and 1298 variables; particularly, *MTHFR *677TT was connected with *MTHFR *1298AA, whereas *MTHFR *1298AC was connected with both *MTHFR *677CC and *MTHFR *677CT variables. Moreover, *TYMS *28bp repeats and 1494 6bp deletion variables were always connected each other; particularly, *TYMS *1494 6bp +/- resulted connected with *TYMS *28bp 2R/3R, *TYMS *1494 6bp +/+ with *TYMS *28bp 2R/2R, and *TYMS *1494 6bp -/- with *TYMS *28bp 3R/3R. BNMN‰, *RFC1 *80GG and *TYMS *28bp 2R/3R resulted to be the three variables connected with the condition of being MDS. On the contrary, *RFC1 *80AA, *MTR *2756AA and *TYMS *28bp 3R/3R resulted to be the three variables connected with the condition of being a control mother.

## Discussion

This study explored the association between 7 polymorphisms in the folate metabolic pathway, chromosome damage in peripheral blood lymphocytes (as BNMN frequency), and the condition of being mother of a DS child with complementary non-linear approaches: supervised neural networks (ANNs), and the semantic connectivity map.

Through TWIST system, we established a consistent possibility to predict the status of being a MDS on the basis of 6 selected variables (Table 3) with 90% specificity, sensitivity and global accuracy (Table 4), this meaning that the selected variables contained specific information on the occurrence of a DS pregnancy. In particular, the BNMN frequency, as well as *RFC1 *80AA, *MTHFR *677TT, *MTR *2756AA, *TYMS *1494 6bp del +/+ and *TYMS *28bp 3R/3R polymorphisms resulted the most important variables for discriminating between MDS and control mothers (Table 3). Most of these variables had been previously associated with DS risk by means of genetic association studies [2].

The present study represents the first attempt to use ANNs to understand the complex relationship between folate metabolism and maternal risk for having a DS child. Though we achieved good results using ANNs for a small dataset, results are not necessarily generalizable to a larger population but need to be validated independently in future studies. A look at Table 1 shows that some of the genotypes, such as for example the *MTHFR *1298CC one, were not present in our cohort while others were present only in a few subjects, indicating the need of further analyses in a larger group. Within this context we made our database available online (see additional file 1) so that other research groups can use the data to increase their own datasets. Moreover, we welcome any further analysis of our database with other methods of forecasting, including logistic regression methods, neural networks, or support vector machines. In addition, present results have been obtained in an Italian case-control cohort and are not generalizable to other populations.

Through the connectivity map (Figure 4) we established the connections between the studied variables and the condition of being MDS or control mother.

Considering individual findings the connectivity map showed several connections already known in the literature, as well as novel ones. Particularly, a connection resulted between *MTHFR *677C>T and 1298A>C polymorphisms, for example among the *MTHFR *677TT and the *MTHFR *1298AA genotypes. This connection can be explained on the basis of a strong linkage disequilibrium existing between the two polymorphisms. MTHFR works as a dimer protein and the combined presence of the T allele at position 677 and of the C allele at position 1298 impairs the stability of the dimer. As a consequence, the 677T allele is in strong linkage with the 1298A one, while the 677T-1298C haplotype is rare and selected negatively [29].

Similarly, the connectivity map (Figure 4) showed a connection between *TYMS *28bp repeats and 1494 6bp deletion polymorphisms. For example, the *TYMS *1494 6bp +/+ genotype resulted connected with the *TYMS *28bp 2R/2R one, and the *TYMS *1494 6bp -/- genotype with the *TYMS *28bp 3R/3R one. Again, linkage disequilibrium is known between these two polymorphisms. A previous study by us, performed in a large cohort of white non-Hispanic Americans, revealed that haplotypes containg both the 6bp deleted (-) allele and the 2R allele are rare [30]. A possible biological explanation could be that the 6bp deletion is likely to impair the stability of the *TYMS *mRNA, while the 2R allele is associated with reduced transcription of the *TYMS *gene. Therefore, their combined presence on the same haplotype could seriously impair TYMS production [30].

The connectivity map (Figure 4) revealed that three variables are closely connected with the condition of being MDS: the *RFC1 *80GG genotype, the BNMN frequency, and the *TYMS *2R/3R genotype. On the contrary, the *RFC1 *80AA genotype, the *TYMS *3R/3R genotype and the *MTR *2756AA genotype, are closely connected to the condition of being a control mother. The association between the *RFC1 *80G allele and increased risk for having a DS child has been often observed in Italian populations. We first reported a possible role for the *RFC1 *80G>A polymorphism, in interaction with *MTHFR *677C>T and 1298A>C variants, in affecting DS risk in Italy, suggesting a causative role for the *RFC1 *80G allele and a protective role for the *RFC1 *80A one [31]. Subsequently, in a larger case-control study, others observed an independent association between the *RFC1 *80GG genotype and increased risk for having a DS child in Italy [32]. They also confirmed the protective and interactive role for the *RFC1 *80A allele previously observed by us [33]. Overall, based on these studies, we concluded that in the Italian population the *RFC1 *80G allele could increase the risk for having a DS child, while the *RFC1 *80A allele could be protective [34]. Interestingly, the connectivity map confirmed this observation, showing a connection between the *RFC1 *80GG genotype and the condition of being MDS, as well as a connection between the *RFC1 *80AA genotype and the condition of being control mother.

The BNMN frequency is another variable connected with the condition of being MDS. Several previous studies by us [11,13,14] revealed a statistically significant increased BNMN frequency in MDS respect to control mothers, leading us to formulate the hypothesis that MDS could have an increased tendency to chromosome malsegregation events during somatic mitotic divisions. More recently others have suggested that women who have a DS child in young age could be characterized by an elevated frequency of mitotic malsegregation events during embryogenesis [12].

The *MTR *2756AA genotype resulted to be connected to the condition of being a control mother as well as to the *MTRR *66AA genotype (Figure 4). As shown in figure 1, MTR and MTRR physically interact during folate metabolism, being MTRR required for the maintenance of MTR in its active state. In 2003, Bosco and co-workers observed association between the *MTR *2756G allele and increased DS risk in Italy, arguing for a protective role for the 2756A allele. They also reported an interaction between *MTR *2756A>G and *MTRR *66A>G polymorphisms in increasing DS risk [35]. We recently observed interactions between *MTR *2756AA and *MTHFR *677TT genotypes in increasing DS risk in Italy [14]. However, as shown by the connectivity map (Figure 4), these two variables are not directly connected, and many other factors might affect their interaction.

A very interesting finding of the present study is in the central role played by *TYMS *polymorphisms in the connectivity map. Indeed, the *TYMS *6bp +/- genotype resulted to be the principal hub of the system, the *TYMS *28bp 2R/3R genotype was connected to the condition of being MDS, and the *TYMS *28bp 3R/3R genotype to that of being a control mother (Figure 4). TYMS shifts the folate metabolic pathway from DNA methylation toward DNA synthesis (Figure 1). Both TYMS and MTHFR compete for the same substrate: 5,10-methyleneTHF, the first for DNA synthesis, the latter for DNA methylation purposes. The connectivity map (Figure 4) shows several connections between *TYMS *and *MTHFR *polymorphisms that can be explained by the following observation: given that both enzymes utilize the same substrate, polymorphisms reducing MTHFR enzyme activity might shift pools of 5,10-methyleneTHF from DNA methylation toward DNA synthesis, whereas polymorphisms affecting TYMS activity might shift the pathway from DNA synthesis toward DNA methylation [30]. We recently observed interaction between *MTHFR *and *TYMS *polymorphisms in increasing DS risk, suggesting that an impaired balance between DNA synthesis and methylation processes could favour chromosome malsegregation events [14]. The results from the present study (Table 3 and Figure 4) seriously argue in favour of a pivotal role for *TYMS *polymorphisms in DS risk.

## Conclusions

In conclusion, the currently available literature suggests that complex interactions between polymorphisms in folate metabolizing genes might account for an increased maternal risk to have a DS child; however, given the complexity of the pathway, those complex interactions cannot be easily understood and none of the so far studied polymorphisms can be used in genetic counselling to predict the maternal risk for having a DS child [2]. The present study identified 6 critical variables that allowed discriminating between MDS and control mothers with 90% sensitivity, specificity and accuracy, and provided important information on the complex biological connections between the studied variables and the two conditions (being MDS or control mothers). The study suggests a link between polymorphisms in the folate metabolic pathway and the risk for a DS pregnancy, indicating complex gene-gene interactions. The overall evidence suggests that further research in the field, such as the addition of other variables, is likely to increase the specificity and sensitivity of the system and could help for the development of screening tools aimed at evaluating the risk for a young woman to have a DS pregnancy.

## Competing interests

The authors declare that they have no competing interests.

## Authors' contributions

FC: conceived and designed the study, supervised all the work related with the collection of genetic and cytogenetic data and all the work in the laboratory, prepared the database for the specific analysis, interpreted the data, wrote the manuscript. EG: carried out the ANNs and PC analysis, was involved with the drafting of the related technical parts in the manuscript including tables and figures, and revised critically the entire manuscript. FM: performed genotyping and helped preparing the database for the specific analysis. LM: supervised the research group, helped drafting and revising critically the entire manuscript. All authors read and approved the final manuscript.

## Pre-publication history

The pre-publication history for this paper can be accessed here:

http://www.biomedcentral.com/1755-8794/3/42/prepub

## Supplementary Material

Additional file 1 **Database**. The database used for analysis in an Excel .csv format. Click here for file

Additional file 2 **Twist System**. The pdf file contains a detailed description of the Twist system. This document already existed as documentation [22,24,25] and is included for clarity. Click here for file

Additional file 3 **Auto-CM System**. The pfd file contains a detailed description of the theory behind Auto-CM system. This document already existed as documentation [26,27] and is included for clarity. Click here for file
