# Supplementary material for: Polymorphisms in folate-metabolizing genes, chromosome damage, and risk of Down syndrome in Italian women: identification of key factors using artificial neural networks
Source: BMC Med Genomics. 2010 Sep 24;3:42. doi: 10.1186/1755-8794-3-42 (PMC2949778; doi:10.1186/1755-8794-3-42)
Supplement: Additional file 2 — Twist System. The pdf file contains a detailed description of the Twist system. This document already existed as documentation [22,24,25] and is included for clarity. [file 1755-8794-3-42-S2.PDF]

Additional file: Twist System

TWIST system is an ensemble of two algorithms: T&T and I.S.

T&T

The "Training and Testing" algorithm (T&T) is based on a population of n ANNs managed by an evolutionary system. In its simplest form, this algorithm reproduces several distribution models of the complete dataset DΓ (one for every ANN of the population) in two subsets ($d_{\Gamma}^{[tr]}$, the Training Set and $d_{\Gamma}^{[ts]}$, the Testing Set). During the learning process each ANN, according to its own data distribution model, is trained on the subsample $d_{\Gamma}^{[tr]}$ and blind-validated on the subsample $d_{\Gamma}^{[ts]}$.

The performance score reached by each ANN in the testing phase represents its "fitness" value (i.e., the individual probability of evolution). The genome of each "network-individual" thus codifies a data distribution model with an associated validation strategy. The n data distribution models are combined according to their fitness criteria using an evolutionary algorithm. The selection of "network-individuals" based on fitness determines the evolution of the population; that is, the progressive improvement of performance of each network until the optimal performance is reached, which is equivalent to the better division of the global dataset into subsets. The evolutionary algorithm mastering this process, named "Genetic Doping Algorithm" (GenD for short), was created at Semeion Research Centre has similar characteristics to a genetic algorithm [22,24,25] but it's able to maintain an inner instability during the evolution, carrying out a natural increase of biodiversity and a continuous "evolution of the evolution" in the population. The elaboration of T&T is articulated in two phases:

1) Preliminary phase: in this phase an evaluation of the parameters of the fitness function that will be used on the global dataset is performed. During this phase an inductor $\Omega_{D_{\Gamma}^{[tr]},A,F,Z}(\cdot)$ is configured, which consists of an Artificial Neural Network with an algorithm (A) Back Propagation standard. For this inductor the optimal configuration to reach the convergence is stabilized at the

end of different training trials on the global dataset D_Γ; in this way the configuration that most "suits" the available dataset is determined: the number of layers and hidden units and some possible generalizations of the standard learning law. The parameters thus determined define the configuration and the initialization of all the individual-networks of the population and will then stay fixed in the following computational phase. Basically, during this preliminary phase there is a fine-tuning of the inductor that defines the fitness values of the population's individuals during evolution.

The accuracy of the ANN performance with the testing set will be the fitness of that individual (that is, of that hypothesis of distribution into two halves of the whole dataset).

2) Computational phase: the system extracts from the global dataset the best training and testing sets. During this phase the individual-network of the population is running, according to the established configuration and the initialization parameters. From the evolution of the population, managed by the GenD algorithm, the best distribution of the global dataset D Γ into two subsets is generated, starting from the initial population of possible solutions $x = \left(D_\Gamma^{[tr]}, D_\Gamma^{[ts]}\right)$. Preliminary experimental sessions are performed using several different initialization and configuration of the network in order to achieve the best partition of the global dataset.

I.S.

Parallel to T&T run I.S. (Input Selection), an adaptive system, which is also based on the evolutionary algorithm GenD, and which is able to evaluate the relevance of the different variables of the dataset in an intelligent way. Therefore it can be considered on the same level as a feature selection technique.

From a formal point of view, I.S. is an artificial organism based on the GenD algorithm and consists of a population of ANN, in which each one carries out a selection of the independent variables on the available database. The elaboration of I.S., as for T&T, is developed in two phases:

1) Preliminary phase: during this phase an inductor $\Omega_{D_{\Gamma}^{[tr]}, A, F, Z}(\cdot)$ is configured to evaluate the parameters of the fitness function. This inductor is a standard Back-Propagation ANN. The parameters configuration and the initialization of the ANNs are carried out with particular care to avoid possible over-fitting problems that can be present when the database is characterized by a high number of variables that describe a low quantity of data. The number of epochs E_0 necessary to train the inductor is determined through preliminary experimental tests.

2) Computational phase: the inductor is active, according to the stabilized configuration and the fixed initialization parameters, to extract the most relevant variables of the training and testing subsets. Each individual-network of the population is trained on the training set $D'^{[tr]}_{\Gamma}$ and tested on the testing set $D'^{[ts]}_{\Gamma}$.

The evolution of the individual-network of the population is based on the algorithm GenD. In the I.S. approach the GenD genome is built by n binary values, where n is the cardinality of the original input space. Every gene indicates if an input variable is to be used or not during the evaluation of the population fitness. Through the evolutionary algorithm, the different "hypotheses" of variable selection, generated by each ANNs of the population, change over time, at each generation: this leads to the selection of the best combination of input variables. As in the T&T systems the genetic operators crossover and mutation are applied on the ANNs population; the rates of occurrence for both operators are self-determinated by the system in adaptive way at each generation.

When the evolutionary algorithm no longer improves its performance, the process stops, and the best selection of the input variables is employed on the testing subset.

In order to improve the speed and the quality of the solutions that have to be optimized, the GenD algorithm makes the evolutionary process of the artificial populations more natural and less centered on the individual liberalism culture.
