# Supplementary material for: Polymorphisms in folate-metabolizing genes, chromosome damage, and risk of Down syndrome in Italian women: identification of key factors using artificial neural networks
Source: BMC Med Genomics. 2010 Sep 24;3:42. doi: 10.1186/1755-8794-3-42 (PMC2949778; doi:10.1186/1755-8794-3-42)
Supplement: Additional file 3 — Auto-CM System. The pfd file contains a detailed description of the theory behind Auto-CM system. This document already existed as documentation [26,27] and is included for clarity. [file 1755-8794-3-42-S3.PDF]

Additional file: Auto-CM System

Auto Contractive Map (Auto-CM) is a special kind of ANN which at variance with classical ANNs which have random initial weight values of the connections start from all connections set up with the same values. So they do not suffer the problem of the symmetric connections.

During training, they develop for each connection only positive values. Therefore, Auto-CM does not present inhibitory relations among nodes, but only different strengths of excitatory connections.

Auto-CM can learn also in hard conditions, that is, when the connections of the main diagonal of the second connections matrix are removed. When the learning process is organized in this way, Auto-CM seems to find a specific relationship between each variable and any other. Consequently, from an experimental point of view, it seems that the ranking of its connections matrix is equal to the ranking of the joint probability between each variable and the others.

After learning process, any input vector, belonging to the training set, will generate a null output vector. So, the energy minimization of the training vectors is represented by a function trough which the trained connections absorb completely the input training vectors. Auto-CM seems to learn to transform itself in a dark body.

At the end of the training phase ($\Delta w_{i,j} = 0$), all the components of the weights vector **v** reach up the same value:

(1) $$\lim_{n \to \infty} v_{i_{(n)}} = C \; .$$

The matrix **w**, then, represents the CM knowledge about all the dataset.

It is possible to transform the **w** matrix also in probabilistic joint association among the variables *m*:

(2) $$p_{i,j} = \frac{w_{i,j}}{\sum_{j=1}^{N} w_{i,j}};$$

(3) $$P(m_j^{[s]}) = \sum_{i}^{N} p_{i,j} = 1$$

The new matrix **p** can be read as the probability of transition from any state-variable to anyone else:

(4) $$P(m_i^{[t]} \middle| m_j^{[s]}) = p_{i,j} .$$

g. At the same time the matrix **w** may be transformed into a non Euclidean distance metric (semi-metric), when we train the CM with the main diagonal of the **w** matrix fixed at value N.

Now, if we consider N as a limit value for all the weights of the **w** matrix, we can write:

(5) $$d_{i,j} = N - w_{i,j}$$

The new matrix **d** is also a squared symmetric matrix where the main diagonal represents the zero distance between each variable from itself.

Auto CM and Minimum Spanning Tree

Equation (5) transforms the squared weights matrix of Auto-CM into a squared matrix of distances among nodes. Each distance between a pair of node becomes, consequently, the weighted edge between these pair of nodes.

At this point, the matrix **d** may be analyzed trough the graph theory.

The Minimum Spanning Tree problem is defined as follows: find an acyclic subset T of E that connects all of the vertices in the graph and whose total weight is minimized, where the total weight is given by:

(6) $$d(T) = \sum_{i=0}^{N-1} \sum_{j=i+1}^{N} d_{i,j}, \forall d_{i,j} .$$

T is called spanning tree, and MST is the T with the minimum sum of its edges weighted.

(7) $$Mst = Min\{d(T_k)\} .$$

Given a undirected Graph G, representing a **d** matrix of distances, with V vertices, completely linked each other, the total number of their edges (E) is:

(8) $$E = \frac{V \cdot (V-1)}{2};$$

And the number of its possible tree is:

(9) $$T = V^{V-2}.$$

Kruskal in the 1956 found out an algorithm able to determinate the MST of any undirected graph in a quadratic number of steps, in the worse case. From conceptual point of view the MST represents the **energy minimization** state of a structure. In fact, if we consider the atomic elements of a structure as vertices of a graph and the strength among them as the weight of each edge, linking a pair of vertex, the MST represents the minimum of energy needed because all the elements of the structure continue to stay together.

In a closed system, all the components tend to minimize the overall energy. So the MST, in specific situations, can represent the most probable state where a system tends to.

To define the MST of a undirected graph, each edge of the graph has to be weighted. The equation (5) shows a way to weight each edge whose nodes are the variables of a dataset and whose weights of a trained AutoCM provides the metrics [26,27].
